# Organoid cultures of MELAS neural cells reveal hyperactive Notch signaling that impacts neurodevelopment

**DOI:** 10.1038/s41419-020-2383-6

**Published:** 2020-03-13

**Authors:** Winanto, Zi Jian Khong, Boon-Seng Soh, Yong Fan, Shi-Yan Ng

**Affiliations:** 1 Institute of Molecular and Cell Biology, A*STAR Research Entities, 138673 Singapore, Singapore; 2 School of Biological Sciences, Nanyang Technological University, 637551 Singapore, Singapore; 3 Department of Biological Sciences, National University of Singapore, 117543 Singapore, Singapore; 4 The Third Affiliated Hospital of Guangzhou Medical University, 510150 Guangzhou, China; 5 Yong Loo Lin School of Medicine (Physiology), National University of Singapore, 117456 Singapore, Singapore; 6 National Neuroscience Institute, 308433 Singapore, Singapore

**Keywords:** Diseases of the nervous system, Induced pluripotent stem cells

## Abstract

Mutations in mitochondrial DNA (mtDNA), typically maternally inherited, can result in severe neurological conditions. There is currently no cure for mitochondrial DNA diseases and treatments focus on management of the symptoms rather than correcting the defects downstream of the mtDNA mutation. Mitochondrial encephalomyopathy, lactic acidosis and stroke-like episodes (MELAS) is one such mitochondrial disease that affects many bodily systems, particularly the central nervous system and skeletal muscles. Given the motor deficits seen in MELAS patients, we investigate the contribution of motor neuron pathology to MELAS. Using a spinal cord organoid system derived from induced pluripotent stem cells of a MELAS patient, as well as its isogenically corrected control, we found that high levels of Notch signaling underlie neurogenesis delays and neurite outgrowth defects that are associated with MELAS neural cultures. Furthermore, we demonstrate that the gamma-secretase inhibitor DAPT can reverse these neurodevelopmental defects.

## Introduction

Mitochondrial encephalomyopathy, lactic acidosis and stroke-like episodes (MELAS) is a multiorgan metabolic disorder that is caused by mutations in the mitochondrial DNA (mtDNA), where the A3243G mutation, affecting the MT-TL1 gene encoding for tRNA^Leu(UUR)^, is most prevalent and accounts for 80% of all MELAS cases. Like most mitochondrial diseases, the severity of MELAS is determined by the heteroplasmy levels of the mtDNA mutation. While most people with MELAS syndrome develop first symptoms before the age of 20, patients with high mutation load tend to present symptoms that occur early in infancy with a history of developmental delays.

Unsurprisingly, the central nervous system and skeletal muscles are the most affected organ systems in MELAS, since neurons and skeletal muscles are well established to utilize mitochondrial respiration to fuel their high metabolic demands. Therefore, symptoms of MELAS include muscle weakness and pain, hypotonia, difficulties in speech and swallowing, lactic acidosis and fatigue, which are also commonly seen in patients with motor neuron diseases (MNDs). While the neuromuscular unit appears to be affected in MELAS, there have been no studies so far investigating the biology of motor neurons or neuromuscular junctions in MELAS, whose pathology could be responsible for the MELAS phenotype.

In this study, we obtained induced pluripotent stem cells (iPSCs) from a patient with severe MELAS, as well as its isogenically corrected control line with the causative A3243G mutation corrected. To investigate motor neuron deficits in MELAS versus its isogenic control, we differentiated these iPSCs into motor neuron progenitors and motor neurons in a standard two-dimensional protocol and as spinal organoids. Our results indicate that while MELAS iPSCs can robustly form motor neuron progenitors, subsequently differentiation towards motor neurons is severely limited. This was due to hyperactive Notch signaling, which prevented neurogenesis. We also found that inhibition of mitochondrial Complex I recapitulated the MELAS phenotype and led to increased Notch signaling, implicating a mitochondrial-Notch crosstalk that is previously unknown in MELAS.

## Results

### MELAS iPSCs differentiate efficiently towards NPCs but fail to generate motor neurons

**Fig. 1:**
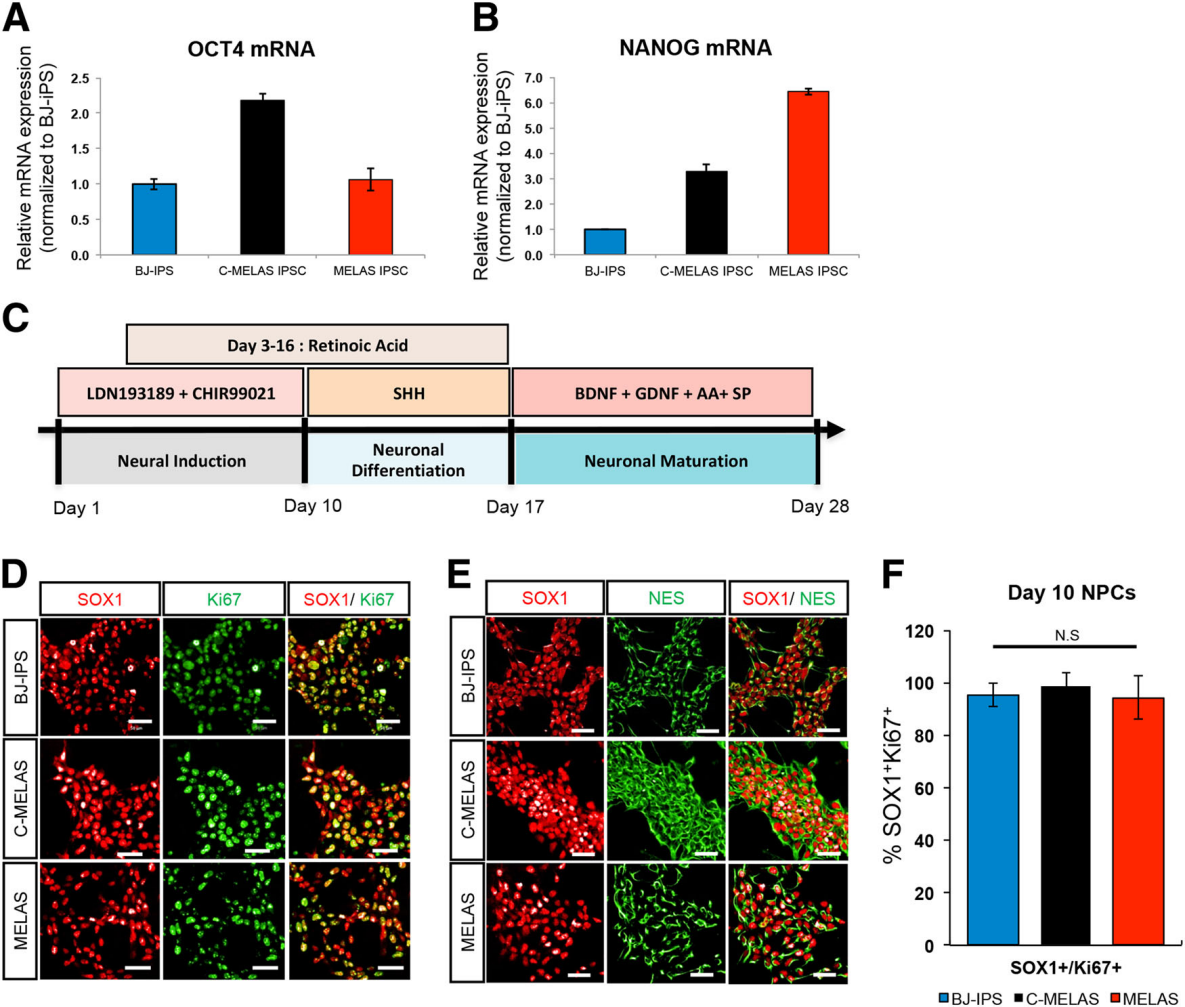
MELAS iPSCs are pluripotent and differentiate efficiently into neural progenitors. **a**, **b** MELAS iPSCs express OCT4 and NANOG mRNA transcripts equivalent to that expressed by wild-type BJ-iPS and isogenic control c-MELAS iPSCs. **c** Schematic of differentiation of iPSCs into neural progenitors (by day 10) and eventually into motor neurons (by day 28). **d**, **e** Immunostaining analyses of day 10 neural progenitors derived from BJ-iPS, c-MELAS and MELAS iPSCs, demonstrating efficient derivation of neural progenitors expressing SOX1, Ki67 and Nestin. **f** Quantification of cells coexpressing SOX1 and Ki67 in respective cultures. Scale bars indicate 100 μm.

Induced pluripotent stem cells derived from MELAS patient with approximately 80% heteroplasmy in the disease-causing A3243G mutation (MELAS iPSC) was subjected to genome correction using MitoTALENs^1^. These isogenically corrected (c-MELAS) iPSCs and a wild-type iPSC line (BJ-iPS) were used as healthy controls. We found that MELAS iPSCs expressed high levels of pluripotent markers OCT4 and NANOG, similar to the control iPSCs (Fig. 1a, b). Since pluripotency of MELAS iPSCs was unaffected, we investigated the neural differentiation potential of these iPSCs. Using protocols established in the laboratory, we differentiated these iPSCs towards motor neurons. To induce differentiation towards motor neurons, iPSCs were subjected to BMP inhibition by LDN-193189, and Wnt signaling activation by high levels of CHIR99021 for 10 days^2,3^ (Fig. 1c). Retinoic acid (RA) was introduced to the cultures from day 3 to caudalize the cultures. Immunostaining analyses at day 10 revealed that MELAS cultures were uniformly differentiated into neural progenitor cells expressing SOX1, Ki67 and Nestin (Fig. 1d, e) with more than 90% of SOX1^+^Ki67^+^ NPCs in the entire culture, similar to that of the isogenically corrected c-MELAS cell line (Fig. 1f). We performed a heteroplasmy determination assay and found that heteroplasmy levels of the day 10 MELAS NPCs remained unchanged at approximately 80%, similar to that of the starting iPSCs (Fig. S1A).

The NPCs derived from MELAS, c-MELAS and BJ-iPS cultures were further differentiated in the presence of RA and Purmorphamine from days 11 to 17, and subsequently supplemented with BDNF and GDNF to promote maturation of motor neurons. While ISL1^+^SMI32^+^ motor neurons were abundant in healthy controls at day 28, MELAS cultures became unviable at days 16–17 (*n* = 8 biological repeats; Fig. S1B). This indicates that while MELAS iPSCs are capable of differentiating into caudalized NPCs, motor neurogenesis was severely affected.

### Spinal organoids derived from MELAS iPSCs show overt neurogenesis defects

**Fig. 2:**
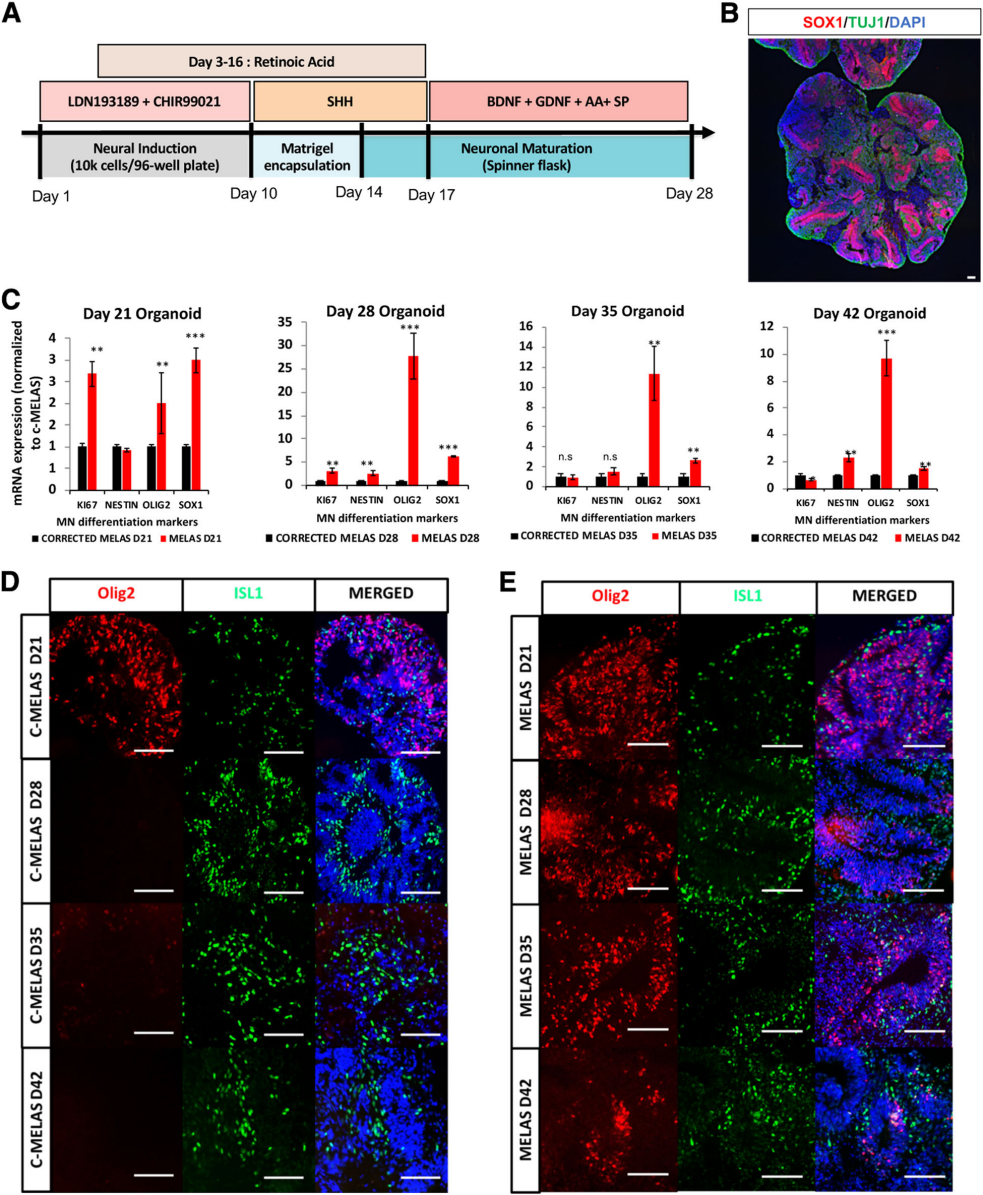
Neural organoids derived from MELAS iPSCs reveal neurodevelopmental defects. **a** Schematic showing derivation of spinal organoids. **b** Representative image of a spinal organoid at day 28, cryosectioned and immunostained with SOX1 and TUJ1 to reveal neural progenitors and neurons. **c** qPCR analyses of days 21, 28, 35 and 42 organoids derived from c-MELAS and MELAS iPSCs demonstrating significant upregulation of OLIG2 and SOX1. **d**, **e** Immunostaining of days 21, 28, 35 and 42 cryosectioned organoids derived from c-MELAS and MELAS iPSCs respectively. Scale bars indicate 100 μm. **p-value < 0.01, ***p-value < 0.001.

To further investigate the neurogenesis defects in MELAS, we derived spinal organoids from MELAS and c-MELAS iPSCs since organoids recapitulate neural developmental processes in vivo^4^. We followed our previous method to generate spinal organoids^3^, where 30,000 cells were seeded in each well of a round-bottom 96-well plate to begin neuralization. Neuralized cells at day 10 were then encapsulated in a droplet of Matrigel and transferred into a spinner flask at day 14 for maturation and growth (Fig. 2a, b). To understand how the mtDNA A3243G mutation affects motor neuron development, we analyzed our spinal organoids at various time points from days 21 to 42. First, qPCR analyses of key motor NPC markers revealed that mRNA levels of OLIG2 and SOX1 were consistently higher in MELAS versus c-MELAS. In particular, OLIG2 expression was more than 25-fold higher in MELAS spinal organoids at day 28, and continued to be approximately tenfold higher than c-MELAS organoids at day 42 (Fig. 2c). As OLIG2 is a motor neuron progenitor marker whose expression diminishes when they differentiate, this suggests that MELAS organoids fail to properly differentiate into motor neurons. To confirm this, we performed immunostaining of OLIG2 in MELAS and c-MELAS organoids at days 21, 28, 35 and 42. In c-MELAS organoids, OLIG2-expressing motor neuron progenitors were observed at day 21 and completely absent at the later time points (Fig. 2d). However, OLIG2-expressing cells were not only abundant in day 21 MELAS organoids, but persisted even until day 42 (Fig. 2e), indicating failure of progenitor differentiation towards motor neurons.

### Notch signaling is elevated in MELAS NPCs

**Fig. 3:**
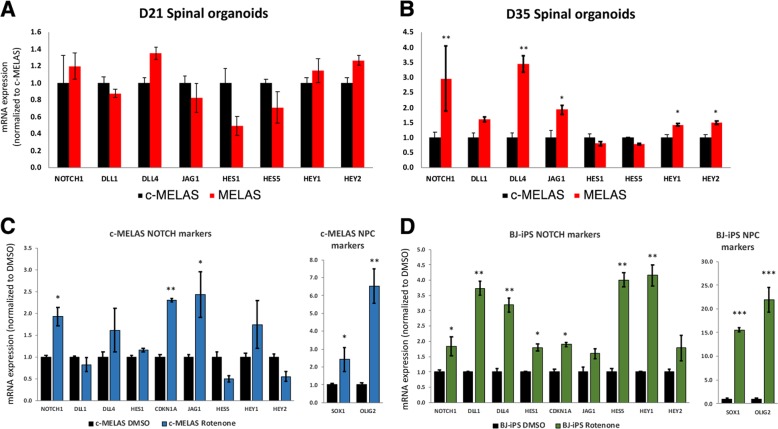
Upregulation of Notch signaling in MELAS organoids. **a**, **b** qPCR analyses of Notch pathway genes in c-MELAS and MELAS organoids at day 21 and day 35 respectively. **c** qPCR analysis of Notch pathway genes and neural progenitor markers SOX1 and OLIG2 in day 35 c-MELAS organoids treated with 0.25μM Rotenone from days 18 to 28. **d** qPCR analysis of Notch pathway genes and neural progenitor markers SOX1 and OLIG2 in day 35 BJ-iPS organoids treated with 0.25μM Rotenone from days 18 to 28. *p-value < 0.05, **p-value < 0.01, ***p-value < 0.001.

Next, we sought to investigate the mechanisms leading to delayed motor neuron differentiation. Since the Notch signaling pathway plays a pivotal role in neurogenesis, we hypothesized that dysregulation of Notch signaling could explain the neurogenesis defects in MELAS. Additionally, Wichterle and colleagues have established that high Notch signaling maintains the population of OLIG2 + motor neuron progenitors in the spinal cord^5^. To investigate if Notch signaling is aberrant in MELAS iPSCs and neural cultures, we analyzed mRNA expression of key components of the Notch pathway in day 21 and day 35 spinal organoids. We found that mRNA expressions of Notch pathway components showed no significant differences at day 21 (Fig. 3a), consistent with the earlier observation that large numbers of OLIG2 progenitors were still present in these early organoids (Fig. 2d, e). However, by day 35, mRNA expressions of Notch receptor *NOTCH1*, NOTCH receptor ligands *DLL1* and *JAG1* as well as the downstream targets *HEY1* and *HES5* were significantly higher in day 35 MELAS compared to controls (Fig. 3b). Therefore, this indicates that the Notch signaling pathway is hyperactive in MELAS neural cultures.

To understand how mitochondrial energetic deficiencies in MELAS could lead to hyperactive Notch signaling, we next investigated effects of mitochondrial complex inhibitors on Notch signaling and neurogenesis. Since mitochondrial Complex I deficiencies have been well-described in MELAS patient-derived cell types^6,7^, we treated the healthy c-MELAS and BJ-iPS organoids with 0.25 μM rotenone, a well-established Complex I inhibitor, from day 18 to day 28. At day 35, gene expression analysis by qPCR was performed, which confirmed upregulation of Notch effector genes including *HES5* and *HEY1*, as well as the NPC markers *SOX1* and *OLIG2* (Fig. 3c, d). This indicates that deficiencies in mitochondrial respiration contributes to elevated Notch signaling and poor differentiation of neural progenitors.

### Notch inhibition corrected neurogenesis and neurite outgrowth defects in MELAS spinal organoids

**Fig. 4:**
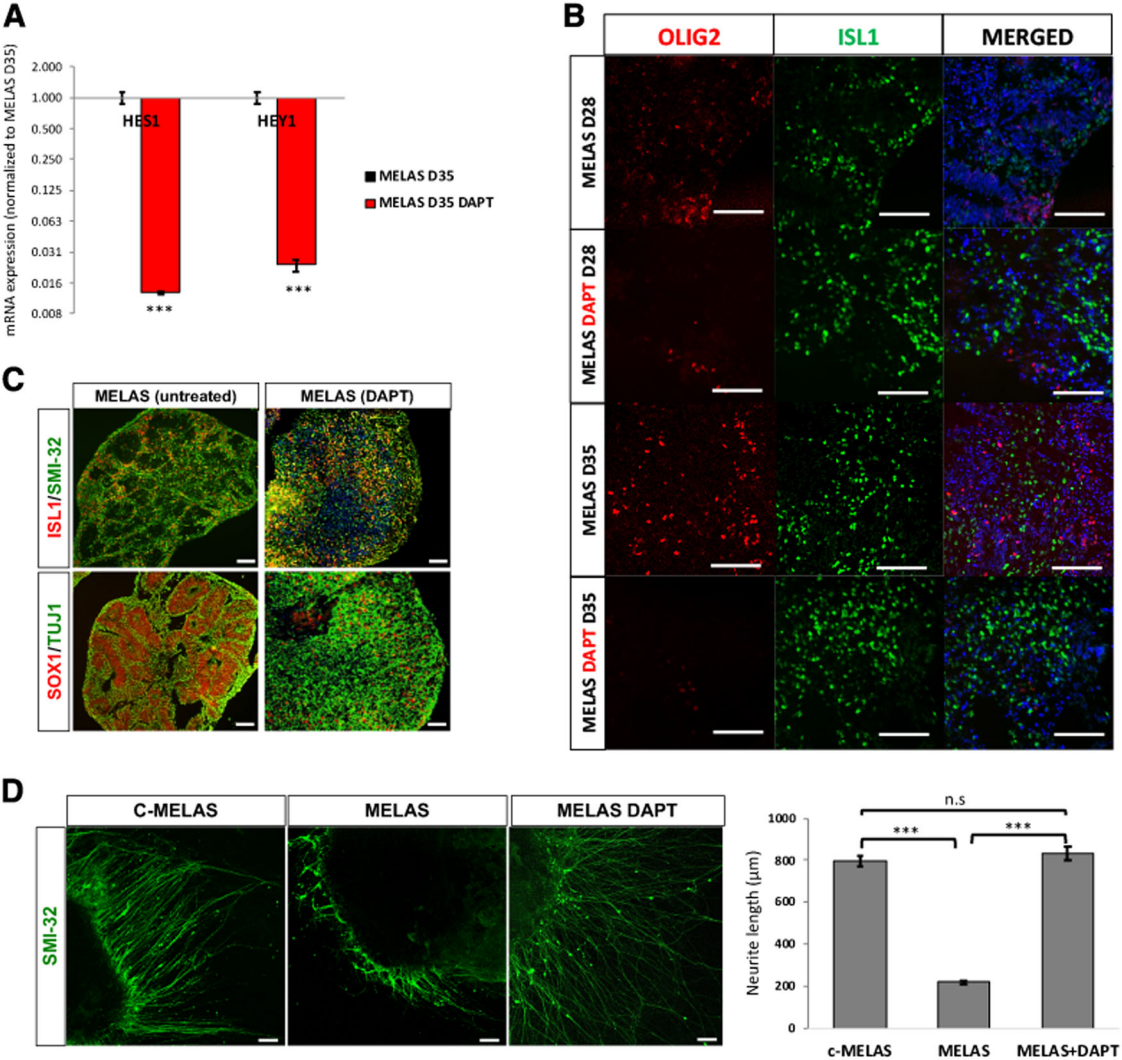
Gamma secretase inhibitor DAPT reverses neurogenesis and neurite outgrowth defects in MELAS organoids. **a** qPCR analysis of Notch effector genes HES1 and HEY1 indicating that DAPT treatment inhibits Notch signaling. **b** MELAS organoids were treated with DMSO or DAPT from days 18 to 28, and immunostaining of OLIG2 and ISL1 was performed at either day 28 or day 35. In the presence of DAPT, the OLIG2 motor neuron progenitor population is reduced while ISL1^+^ motor neuron population is increased. **c** Immunostaining of cryosectioned organoids derived from MELAS iPSCs revealed changes in the cyto-architecture after DAPT treatment. **d** Day 21 organoids were seeded onto Matrigel-coated plates and allowed to attach and extend their neurites for 7 days. On day 28, cultures were fixed and immunostaining for motor neuron axon marker SMI-32 was performed. Neurite lengths were then measured using ImageJ. Scale bars indicate 100 μm. ***p-value < 0.001.

It has been well established that Notch signaling maintains the stem cell identity in NPCs and inhibition of Notch is necessary for neuronal differentiation^5,8,9^. We postulate that MELAS NPCs are not able to differentiate efficiently due to constitutively high Notch signaling. Therefore, to investigate if inhibition of Notch pathway would correct the neurogenesis defects in MELAS NPCs, we treated MELAS organoids with 2.5 μM DAPT from day 18 to day 28. As expected, DAPT treatment resulted in significant reduction of downstream Notch targets *HEY1* and *HES1* (Fig. 4a). Immunostaining of MELAS organoids treated with DAPT was also performed, which revealed the significant reduction of OLIG2^+^ motor neuron progenitors at day 28, which were almost completely depleted by day 35 (Fig. 4b). This was similar to c-MELAS organoids, where OLIG2-expressing cells were almost undetectable by day 28 (Fig. 2e). In addition, the depletion of SOX1^+^ neural rosette structures (Fig. 4c), along with increased numbers of ISL1^+^ motor neurons in DAPT-treated organoids (Fig. 4b, c), demonstrated that the neurogenesis defect in MELAS organoids was effectively reversed using DAPT.

Notch signaling has also been previously shown to inhibit neurite outgrowth in neurons^10–12^. Therefore, we investigated the neurite outgrowth of c-MELAS and MELAS organoids by plating the organoids on Matrigel-coated plates and measuring lengths of neurites stained with SMI-32. Consistent with phenotypes of high Notch signaling, neurites from MELAS organoids were shorter than its isogenic c-MELAS organoids. With the same DAPT treatment paradigm from day 18 to 28, we demonstrate that the neurite outgrowth defect was corrected (Fig. 4d). Taken together, these sets of experiments indicate that use of a Notch pathway inhibitor such as DAPT was effective in reversing the neurodevelopmental defects associated with MELAS neurons.

## Discussion

MELAS is traditionally classified as a neurodegenerative disorder, and it has been well established that impairments of mitochondrial respirations can lead to neuronal death. However, symptoms such as delayed development and growth in early-onset patients or psychiatric conditions in a subset of adult-onset MELAS patients suggest that the mtDNA A3243G mutation also leads to neurodevelopmental deficits that remain undescribed to date. To investigate neurodevelopment in MELAS, we made use of patient-derived iPSCs and an isogenically corrected control (c-MELAS), and differentiated these iPSCs towards NPCs and eventually neurons. One interesting observation was that motor neurons were not viable and cannot be derived in our conventional 2D differentiation protocol, but are reproducibly generated in our organoid model. This study adds on to the increasing body of work that indicates neural organoids to be the ideal cell culture model for modeling neural development.

One of the key findings in our study was that defects in mitochondrial respiration, caused either by Complex I inhibition or mtDNA A3243G mutation, would result in elevated Notch signaling. Recent evidence has suggested that mitochondrial fission drives Notch signaling^13^, and the “activated” form of Notch, Notch Intracellular Domain (NICD), has a function in the mitochondria to preserve mitochondrial integrity and promote cell survival^14^, forming a feedback loop. Fragmented mitochondrial networks have been described for MELAS cells^15^, providing more support for this hypothesis. In MELAS, we found that hyperactive Notch signaling results in delayed neuronal differentiation, and that the gamma secretase inhibitor DAPT reversed the neurodevelopmental and neurite outgrowth defects in MELAS organoids.

In summary, our report is one of few studies providing clear evidence that neurodevelopmental defects are intrinsic to MELAS, and implies that motor deficits in MELAS patients is a result of a combination of muscular and motor neuron pathologies. Similarly, neurodevelopmental defects in the cortical neurons could explain developmental delays and psychiatric manifestations that some MELAS patients display^16^.

## Experimental procedures

### Culture, differentiation and quality control of human pluripotent stem cells

Wild-type BJ fibroblast-derived iPSCs (BJ-iPS), patient-derived MELAS iPSCs and corrected MELAS isogenic control iPSCs (c-MELAS) were maintained feeder-free on Matrigel-coated plates in StemMACS^TM^ iPS-Brew media (Miltenyi Biotec). Routine passaging of iPSCs was performed using ReLeSR^TM^ (STEMCELL Technologies) at 1:6 split ratio every week. Pluripotent stem cells were differentiated towards spinal motor neurons following established protocol published previously^3^. To generate spinal organoids, iPSCs colonies were first dissociated into single cells and seeded at 30,000 cells per well in a 96-well low attachment round-bottom plate. At day 10 of differentiation, embryoid bodies were then encapsulated in 15 μl Matrigel droplets before maturing them in spinner flask at day 14. Organoids were routinely harvested at days 21, 28, 25 and 42 for downstream analysis.

Deep sequencing of the entire mtDNA genome was performed for MELAS and c-MELAS iPSCs previously^1^ to confirm the heteroplasmic levels of the A3243G mutation. No other deleterious mutations were found. Subsequently, heteroplasmy was determined for MELAS and c-MELAS iPSCs every 5–6 passages using the restriction fragment length polymorphism (RFLP) method as described previously^1^.

### Organoid analyses

For immunostaining, at least three organoids were fixed with 4% paraformaldehyde overnight, and serially dehydrated with 15 and 30% sucrose overnight on the second and third day respectively. Dehydrated organoids were subsequently embedded in OCT compound for cryosectioning at 10 µm.

For RNA analysis, at least three organoids were pooled together in each biological replicate. Three biological replicates were performed. The organoids were dissolved in Trizol reagent (Life Technologies) for RNA extraction.

### Immunostaining, image acquisition and image analysis

Cells were fixed using 4% paraformaldehyde for 15 min, permeabilized with 0.1% Triton X-100 (for adherent cultures) or 0.5% Triton X-100 (for organoids) for 20 min and blocked in buffer containing 5% FBS and 1% BSA for 2 h at room temperature. Primary antibodies were diluted in blocking buffer and incubated overnight at 4 °C. The following antibodies (and their dilutions) were used in this study: Rabbit SOX1 (1:750, ab87775), mouse Nestin (1:2000, ab22035), mouse Ki67 (1:1500, Cell signaling tech 9449S), rabbit ISL1 (1:1500, ab203406), mouse SMI-32 (1:2000, Calbiochem NE-1023), mouse TUJ1 (1:1000, Biolegend 801202). The respective secondary antibodies (Molecular Probes, Invitrogen) were diluted 1:1500 in blocking buffer and incubated at room temperature, in the dark, for 90 min. DAPI was used at 0.5 μg/ml to visualize cellular nuclei.

For adherent cells in 96-well plates, fluorescence images were obtained using Phenix high content imaging microscope (Perkin Elmer) at ×20 magnification. Image analysis was performed using Columbus (Perkin Elmer). Organoid sections were taken using a fluorescence microscope (Nikon) at ×10 magnification.

### RNA extraction and expression analysis

Total RNA was extracted using RNeasy Mini Kit (Qiagen) according to the manufacturer’s instruction. For quantitative PCR (qPCR), 500 ng RNA per sample was reverse transcribed using high-capacity cDNA Reverse Transcription Kit (Applied Biosystems, USA). Quantitative PCR (qPCR) was performed on the QuantStudio5 Real-Time PCR System using FAST SYBR Master Mix (all from Applied Biosystems). Gene expressions were normalized to HPRT. A list of the qPCR primers used is provided in Table S1.

### DAPT and Rotenone treatments

On day 18 of organoid culture, 5−6 BJ-RIPs, MELAS and C-MELAS organoids were harvested and transferred onto ultra-low attachment six-well plates. Maturation media was supplemented to the culture together with 2.5 μM DAPT (Stem Cell Technologies) or 0.25 μM Rotenone (Sigma). Organoids cultures were placed on an orbital shaker in the cell culture incubator from day 18 to day 28 of differentiation before fixation and cryosectioning at the respective time points.

### Data statistical analysis

Sample size calculations were not performed. Experiments were performed in biological triplicates, with at least three technical replicates each. Within each cell line, samples were randomized for experiments. Data were only excluded from failed experiments. Investigators were not blinded to iPSC genotype and/or treatment. Data reported for cellular and molecular assays are not subjective but based on quantitative morphology, gene expression and cell counts. Statistical analyses of biological replicates were done using GraphPad Prism 7.02 and Microsoft Excel 2017. Biological significance was calculated by means of an unpaired Student’s *t* test. Error bars represent mean ± standard deviation. * indicates *p* values less than 0.05; ** indicates *p* values less than 0.01; *** indicates *p* values less than 0.001.

## Supplementary information

Supplemental Information

Supplemental Figure

Supplemental Table
