# Supplementary material for: Organoid cultures of MELAS neural cells reveal hyperactive Notch signaling that impacts neurodevelopment
Source: Cell Death Dis. 2020 Mar 13;11(3):182. doi: 10.1038/s41419-020-2383-6 (PMC7069952; doi:10.1038/s41419-020-2383-6)
Supplement: Supplementary file 1 — Supplemental Information [file 41419_2020_2383_MOESM1_ESM.docx]

Supplemental Information

**Organoid Cultures of MELAS Neural Cells Reveal Hyperactive Notch Signaling that Impacts Neurodevelopment**

Winanto^1^, Zi Jian Khong^1,2^, Boon-Seng Soh^1,3,4,#^, Yong Fan^4,#^, Shi-Yan Ng^1,4,5,6#^

^1^ Institute of Molecular and Cell Biology, A*STAR Research Entities, Singapore 138673

^2^ School of Biological Sciences, Nanyang Technological University, Singapore 637551

^3^ Department of Biological Sciences, National University of Singapore, Singapore 117543

^4^ The Third Affiliated Hospital of Guangzhou Medical University, 510150 Guangzhou, China

^5^ Yong Loo Lin School of Medicine (Physiology), National University of Singapore, Singapore 117456

^6^ National Neuroscience Institute, Singapore 308433

^#^Correspondence to Shi-Yan Ng ([syng@imcb.a-star.edu.sg](mailto:syng@imcb.a-star.edu.sg)), Yong Fan ([yongfan011@gzhmu.edu.cn](mailto:yongfan011@gzhmu.edu.cn)) and Boon-Seng Soh ([bssoh@imcb.a-star.edu.sg](mailto:bssoh@imcb.a-star.edu.sg))

**SUPPLEMENTAL METHODS**

**Heteroplasmy level determination**

The heteroplasmy of level of different cell lines was obtained by extracting total genomic DNA using Nucleospin Tissue kit (Macherey-Nagel) according to manufacturer protocols. Subsequently, the mtDNA 3243 locus was amplified using following primers (forward 5’-CCTCGGAGCAGAACCCAACCT; reverse, 5’-CGAAGGGTTGTAGTAGCCCGT) which yield 634 bp PCR products. PCR products were digested with ApaI which produced two PCR fragments of 424bp and 210bp. PCR products were then run on 1.2% agarose gel at 100 volts for an hour. The gel was imaged using ChemiDoc and further analyzed using ImageJ by calculating the intensity of individual PCR products compared to its negative control.

**SUPPLEMENTAL FIGURES**


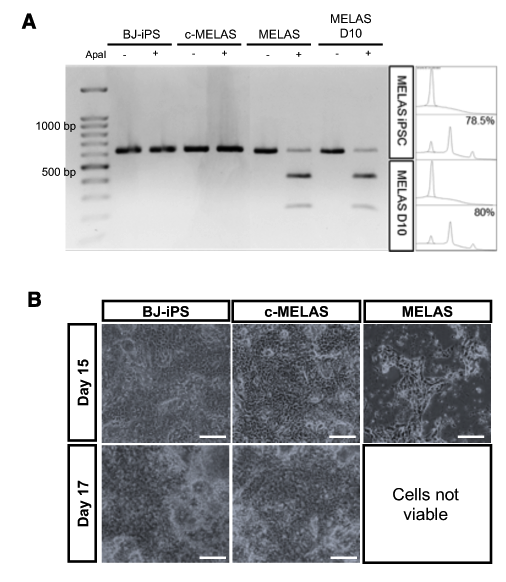


**Figure S1: MELAS iPSCs with high heteroplasmy do not differentiate into viable neurons.** (A) Heteroplasmy determination by ApaI differential digestion of mitochondrial DNA PCR products showing that MELAS iPSCs had 78.5% heteroplasmic load of mutant DNA, which did not change substantially upon differentiation into neural progenitors at day 10. (B) Subsequent differentiation of MELAS day 10 neural progenitors did not result in viable neurons.

**Table S1: List of qPCR primers used in this study**

| S/N | Target Genes | 5' to 3’ Primer Sequence | |
| --- | --- | --- | --- |
| 1 | OCT4 | F | GGAGAGCAACTCCGATGG |
|  |  | R | TTGATGTCCTGGGACTCCTC |
| 2 | NANOG | F | ATGCCTCACACGGAGACTGT |
|  |  | R | AGGGCTGTCCTGAATAAGCA |
| 3 | Ki67 | F | CGTAGCAGCACAGAAAT |
|  |  | R | TGATGGTTGAGGTCGTTCCTTGATG |
| 4 | NESTIN | F | CAGCGTTGGAACAGAGGTTGG |
|  |  | R | TGGCACAGGTGTCTCAAGGGTAG |
| 5 | SOX1 | F | GCGGAAAGVGTTTTTCTTG |
|  |  | R | TAATCTGACTTCTCCTCC |
| 6 | OLIG2 | F | ATGCACGACCTCAACATCGCCA |
|  |  | R | ACCAGTCGCTTCATCTCCTCCA |
| 7 | HPRT | F | TATGGCGACCCGCAGCCCT |
|  |  | R | CATCTCGAGCAAGACGTTCAG |
| 8 | ACTIN B | F | CCAACCGCGAGAAGATGA |
|  |  | R | CCAGAGGCGTACAGGGATAG |
| 10 | GAPDH | F | AGCCACATCGCTCAGACAC |
|  |  | R | GCCCAATACGACCAAATCC |
| 11 | NOTCH1 | F | AAGCTGCATCCAGAGGCAAAC |
|  |  | R | TGGCATACACACTCCGAGAACAC |
| 12 | NOTCH2 | F | AAGGAACCTGCTTTGATGACA |
|  |  | R | CAGGGAGCCAATACTGTCTGA |
| 13 | NOTCH3 | F | CCTAGTCCTGGCTCCGAAC |
|  |  | R | GAGCCGCTTGTCAATCTCC |
| 14 | NOTCH4 | F | ACTGCCTCTGTCCTGATGGA |
|  |  | R | AACCCACGTCACACACACAT |
| 15 | JAG1 | F | TGCCAAGTGCCAGGAAGT |
|  |  | R | GCCCCATCTGGTATCACACT |
| 16 | JAG2 | F | TGGGACTGGGACAACGATAC |
|  |  | R | ATGCGACACTCGCTCGAT |
| 17 | DLL1 | F | GATGTGATGAGCAGCATGGA |
|  |  | R | CCATGGAGACAGCCTGGATA |
| 18 | DLL3 | F | CACTCAACAACCTAAGGACGCAG |
|  |  | R | GAGCGTAGATGGAAGGAGCAGA |
| 19 | DLL4 | F | GGCCAACTATGCTTGTGAATGTC |
|  |  | R | ACCTCGGTTCAGGCACTGTC |
| 20 | HES1 | F | GGAAATGACAGTGAAGCACCTCC |
|  |  | R | GAAGCGGGTCACCTCGTTCATG |
| 21 | HEY1 | F | CTGGCTATGGACTATCGGAGT |
|  |  | R | GACCAGGCGAACGAGAAGC |
| 22 | ADAM10 | F | CTG CCCAGCATCTGACCCTAA |
|  |  | R | TTGCCATCAGAACTGGCACAC |
| 23 | PSEN2 | F | CAGCGCAACTATGAACTTGGAG |
|  |  | R | CATCCTGGGAGAAAGAACAGATC |
| 24 | PSEN1 | F | GCAGTATCCTCGCTGGTGAAGA |
|  |  | R | CAGGCTATGGTTGTGTTCCAGTC |
| 25 | JAK2 | F | CCAGATGGAAACTGTTCGCTCAG |
|  |  | R | GAGGTTGGTACATCAGAAACACC |
| 26 | HES5 | F | CCGGTGGTGGAGAAGATGCG |
|  |  | R | GCGACGAAGGCTTTGCTGTG |
| 27 | HEY2 | F | TGAGAAGACTTGTGCCAACTGCT |
|  |  | R | CCCTGTTGCCTGAAGCATCTTC |
| 28 | CDKN1A | F | AGGTGGACCTGGAGACTCTCAG |
|  |  | R | TCCTCTTGGAGAAGATCAGCCG |
