# Supplementary figures and images for: Organoid cultures of MELAS neural cells reveal hyperactive Notch signaling that impacts neurodevelopment
Source: Cell Death Dis. 2020 Mar 13;11(3):182. doi: 10.1038/s41419-020-2383-6 (PMC7069952; doi:10.1038/s41419-020-2383-6)

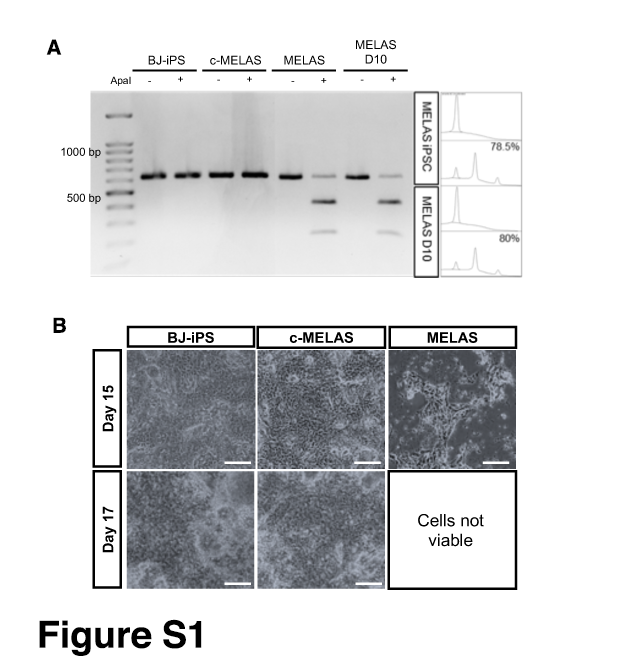

Supplement: Supplementary file 2 — Supplemental Figure [file 41419_2020_2383_MOESM2_ESM.tif]
