# Supplementary material for: Organoid cultures of MELAS neural cells reveal hyperactive Notch signaling that impacts neurodevelopment
Source: Cell Death Dis. 2020 Mar 13;11(3):182. doi: 10.1038/s41419-020-2383-6 (PMC7069952; doi:10.1038/s41419-020-2383-6)
Supplement: Supplementary file 3 — Supplemental Table [file 41419_2020_2383_MOESM3_ESM.docx]

**Supplementary Table S1: List of qPCR primers used in this study**

| S/N | Target Genes | 5' to 3’ Primer Sequence | |
| --- | --- | --- | --- |
| 1 | OCT4 | F | GGAGAGCAACTCCGATGG |
|  |  | R | TTGATGTCCTGGGACTCCTC |
| 2 | NANOG | F | ATGCCTCACACGGAGACTGT |
|  |  | R | AGGGCTGTCCTGAATAAGCA |
| 3 | Ki67 | F | CGTAGCAGCACAGAAAT |
|  |  | R | TGATGGTTGAGGTCGTTCCTTGATG |
| 4 | NESTIN | F | CAGCGTTGGAACAGAGGTTGG |
|  |  | R | TGGCACAGGTGTCTCAAGGGTAG |
| 5 | SOX1 | F | GCGGAAAGVGTTTTTCTTG |
|  |  | R | TAATCTGACTTCTCCTCC |
| 6 | OLIG2 | F | ATGCACGACCTCAACATCGCCA |
|  |  | R | ACCAGTCGCTTCATCTCCTCCA |
| 7 | HPRT | F | TATGGCGACCCGCAGCCCT |
|  |  | R | CATCTCGAGCAAGACGTTCAG |
| 8 | ACTIN B | F | CCAACCGCGAGAAGATGA |
|  |  | R | CCAGAGGCGTACAGGGATAG |
| 10 | GAPDH | F | AGCCACATCGCTCAGACAC |
|  |  | R | GCCCAATACGACCAAATCC |
| 11 | NOTCH1 | F | AAGCTGCATCCAGAGGCAAAC |
|  |  | R | TGGCATACACACTCCGAGAACAC |
| 12 | NOTCH2 | F | AAGGAACCTGCTTTGATGACA |
|  |  | R | CAGGGAGCCAATACTGTCTGA |
| 13 | NOTCH3 | F | CCTAGTCCTGGCTCCGAAC |
|  |  | R | GAGCCGCTTGTCAATCTCC |
| 14 | NOTCH4 | F | ACTGCCTCTGTCCTGATGGA |
|  |  | R | AACCCACGTCACACACACAT |
| 15 | JAG1 | F | TGCCAAGTGCCAGGAAGT |
|  |  | R | GCCCCATCTGGTATCACACT |
| 16 | JAG2 | F | TGGGACTGGGACAACGATAC |
|  |  | R | ATGCGACACTCGCTCGAT |
| 17 | DLL1 | F | GATGTGATGAGCAGCATGGA |
|  |  | R | CCATGGAGACAGCCTGGATA |
| 18 | DLL3 | F | CACTCAACAACCTAAGGACGCAG |
|  |  | R | GAGCGTAGATGGAAGGAGCAGA |
| 19 | DLL4 | F | GGCCAACTATGCTTGTGAATGTC |
|  |  | R | ACCTCGGTTCAGGCACTGTC |
| 20 | HES1 | F | GGAAATGACAGTGAAGCACCTCC |
|  |  | R | GAAGCGGGTCACCTCGTTCATG |
| 21 | HEY1 | F | CTGGCTATGGACTATCGGAGT |
|  |  | R | GACCAGGCGAACGAGAAGC |
| 22 | ADAM10 | F | CTG CCCAGCATCTGACCCTAA |
|  |  | R | TTGCCATCAGAACTGGCACAC |
| 23 | PSEN2 | F | CAGCGCAACTATGAACTTGGAG |
|  |  | R | CATCCTGGGAGAAAGAACAGATC |
| 24 | PSEN1 | F | GCAGTATCCTCGCTGGTGAAGA |
|  |  | R | CAGGCTATGGTTGTGTTCCAGTC |
| 25 | JAK2 | F | CCAGATGGAAACTGTTCGCTCAG |
|  |  | R | GAGGTTGGTACATCAGAAACACC |
| 26 | HES5 | F | CCGGTGGTGGAGAAGATGCG |
|  |  | R | GCGACGAAGGCTTTGCTGTG |
| 27 | HEY2 | F | TGAGAAGACTTGTGCCAACTGCT |
|  |  | R | CCCTGTTGCCTGAAGCATCTTC |
| 28 | CDKN1A | F | AGGTGGACCTGGAGACTCTCAG |
|  |  | R | TCCTCTTGGAGAAGATCAGCCG |
